# Analysis of the impact of the SARS-CoV-2 infection on the pediatric population hospitalized during the pandemic in the Greater Paris University Hospitals

**DOI:** 10.3389/fped.2023.1044352

**Published:** 2023-02-27

**Authors:** Michaela Semeraro, Pierre Pinson, Margaux Populaire, Mourad Dellagi, Mehdi Oualha, Nathanael Beeker, Hélène Chappuy

**Affiliations:** ^1^ Centre D'Investigation Clinique-Unité de Recherche Clinique, Hôpital Universitaire Necker Enfants-Malades, GH Paris Centre, Paris, France; ^2^ EA7323 Pediatric and Perinatal Drug Evaluation and Pharmacology, Université Paris Cité, Assistance Publique-Hôpitaux de Paris (AP-HP), Paris, France; ^3^ Unité de Recherche Clinique, Hôpital Cochin, Paris, France; ^4^ Pediatric Intensive Care Unit, Necker-Enfants-Malades University Hospital, Assistance Publique-Hôpitaux de Paris, Paris University, EA7323, Paris, France; ^5^ Service D'Urgences Pédiatriques, Hôpital Necker - Enfants Malades, Groupe Hospitalier AP-HP.Centre, EA7323, Université Paris Cité, Paris, France

**Keywords:** data warehouse, SARS-CoV-2, children, PIMS, MIS-C multisystem inflammatory syndrome in children, hospitalized child

## Abstract

**Background:**

The clinical characteristics, disease progression and outcome in children affected by severe acute respiratory syndrome coronavirus 2 (SARS-CoV-2) infection appear significantly milder compared to older individuals. Nevertheless, the trends in hospitalization and clinical characteristics in the pediatric population seem to be different over time across the different epidemic waves.

**Objective:**

Our aim was to understand the impact of the different COVID-19 variants in the pediatric population hospitalized in the Pediatric Departments of the Public Hospital in the Greater Paris area by the analysis performed with the Assistance Publique-Hopitaux de Paris (AP-HP) Health Data Warehouse.

**Methods:**

This is a retrospective cohort study including 9,163 patients under 18 years of age, hospitalized from 1 March 2020 to 22 March 2022, in the Paris area, with confirmed infection by SARS-CoV-2. Three mutually exclusive groups with decreasing severity (Pediatric Inflammatory Multisystem Syndrome (PIMS), symptomatic infection, mild or asymptomatic infection) were defined and described regarding demography, medical history, complication of the SARS-CoV-2 infection, and treatment during admission. Temporal evolution was described by defining three successive waves (March–September 2020, October 2020–October 2021, and November 2021–March 2022) corresponding to the emergence of the successive variants.

**Results:**

In the study period, 9,163 pediatric patients with SARS-CoV-2 infection were hospitalized in 21 AP-HP hospitals. The number of patients with SARS-CoV-2 infection increased over time for each wave of the pandemic (the mean number of patients per month during the first wave was 332, 322 during the 2nd, and 595 during the third wave). In the medical history, the most associated concomitant disease was chronic respiratory disease. Patients hospitalized during the third wave presented a higher incidence of pulmonary involvement (10.2% compared to 7% and 6.5% during the first and second waves, respectively). The highest incidence of PIMS was observed during the first and second waves (4.2% in the first and second waves compared to 2.3% in the 3rd wave).

**Discussion:**

This analysis highlighted the high incidence of hospitalized children in the Greater Paris Area during the third wave of SARS-CoV-2 pandemic corresponding to the Omicron Covid-19 variant, which is probably an expression of a concomitant SARS-CoV-2, while a decreased incidence of PIMS complication was observed during the same period.

## Introduction

The severe acute respiratory syndrome coronavirus 2 (SARS-CoV-2) pandemic was declared by the World Health Organization on 11 March 2020. Since this time, several waves of infections related to specific variants of the SARS-CoV-2 have emerged. These variants are due to the virus's acquired additional advantageous mutations, potentially altering transmissibility, severity, and escape from natural or vaccine-derived immunity.

Compared to adults, SARS-CoV-2 infection in the children population presented different characteristics and courses (1–3). First data from China suggested that pediatric COVID-19 infections might be less severe than cases in adults, and children might experience different symptoms than adults (4).

In this regard, specific characteristics and consequences of the pandemic in young patients have been described. First of all, since the beginning of the pandemic, several countries reported cases of a rare Pediatric Inflammatory Multisystem Syndrome temporally associated with SARS-CoV-2 (PIMS-TS) also named Multisystem Inflammatory Syndrome in Children (MIS-C), characterized by Kawasaki-like symptoms and toxic shock syndrome (5–7).

The impact of the variants on the clinical courses in adult patients is well known (i.e., the Omicron-variant surge has been characterized by a mild severity of the disease compared to the Delta variant infection (8)), but little is known about the different patterns of clinical characteristics and outcomes in pediatric patients according to the different surges of infection.

The aim of this study was to understand the impact of the COVID-19 variants in the pediatric population presenting with SARS-CoV2 infection hospitalized in the Public Hospitals of the Paris metropolitan area over the different surges of the pandemic and particularly during the last Omicron variant.

## Methods

### Data source

The Assistance Publique–Hôpitaux de Paris (AP-HP) is the largest hospital entity in Europe, with 39 hospitals (22,474 beds) mainly located in the Greater Paris area.

This retrospective cohort study was conducted using the Assistance Publique-Hopitaux de Paris (AP-HP) Health Data Warehouse (Entrepôt de Données de Santé (EDS), https://eds.aphp.fr/; AP-HP Covid Clinical Data Warehouse (CDW)). This data warehouse contains electronic health records (EHRs) of all inpatients from the 39 greater Paris university hospitals. The clinical data repository has received authorization from the French Data Protection Authority (Commission Nationale de l'Informatique et des Libertés, number 1980120).

As such, the members of the AP-HP Covid CDW initiative contributed to the design and implementation of the database but did not participate in the analysis or writing of this report. A complete listing of members can be found at https://eds.aphp.fr/covid-19.

This analysis follows recommendations provided by the Reporting of Studies Conducted Using Observational Routinely Collected Health Data Statement (9).

This study was approved by the institutional review board (N° CSE: CSE 20–48_COVIDPed; AP-HP CDW Scientific and Ethics Committee number IRB00011591) of the scientific and ethical committee of the AP-HP. All subjects included in this study were informed about the reuse of their data for research, and subjects who objected to the reuse of their data were excluded from this study in accordance with French legislation.

### Patients and study groups

We retrospectively retrieved the data of all patients with COVID-19 infection hospitalized in one of the AP-HP hospitals between March 2020 and March 2022 from the EDS-COVID database.

Inclusion criteria were:

1. Full hospitalization in one of the AP-HP pediatric departments between 1 March 2020 and 22 March 2022.
2. SARS-CoV-2 positive diagnosis and/or PIMS diagnosis according to the International Classification of Diseases (ICD-10 code) related to the hospitalization or positive SARS-CoV2 PCR test between 5 days prior to the start of the hospitalization and 15 days after.
3. Minor patient (<18 years old) at the time of hospitalization.

Comorbidities were extracted from ICD-10 codes used during the previous hospitalizations of each patient if available. Outcomes and treatments during hospitalization were obtained from the current hospitalization codes (ICD-10 and technical treatment French codification-CCAM). PIMS outcome code screening was extended up to 28 days after the start of hospitalization considering the delay between SARS-CoV-2 infection and PIMS described in the literature (10). Correspondence between variables and codes is presented in Supplementary Material Table S2.

Age at admission, sex, intensive care unit (ICU) admission, and death during hospitalization were obtained from the hospital administrative data.

Data are presented as mean (standard deviation) and numbers (%).

The study population comprised three retrospective cohorts:

Cohort 1: Patients who had their first SARS-CoV-2 infections or PIMS between March 2020 to September 2020 corresponding to the emergent first historical variant cohort.
Cohort 2: Patients hospitalized during the second wave, October 2020 to October 2021, including the emergent Delta and Alpha/Beta/Gamma variant cohorts in addition to the cohort of patients without a determined variant type.
Cohort 3: Patients hospitalized during the third wave, November 2021 to February 2022, corresponding to the Delta and Omicron variant cohorts and patients with a non-determined variant.

For the analysis of the population, we defined three mutually excluded groups with decreasing severity. The first group was represented by pediatric patients with PIMS diagnosis or acute myocarditis. The second group was represented by pediatric patients with a principal diagnosis of SARS-CoV-2 infection, and the third group was defined by a positive PCR test or secondary diagnosis of SARS-CoV2, referred to as clinically mild or asymptomatic infection group.

To assess the differences between waves, we performed chi-squared tests, first on the PIMS distribution within the population, then on the symptomatic SARS-CoV2 within the population, with a significance level of 5%. For each cohort, we performed chi-squared or one-way ANOVA test when appropriate, to compare covariates across waves.

## Results

A total of 9,163 pediatric patients hospitalized across 21 APHP hospitals with SARS-CoV-2 infection were included between March 2020 and March 2022: 2,325 patients were included in cohort 1 (mean number of patients detected per month: 332), 3,861 were included in cohort 2 (mean number of patients detected per month: 322), and 2,977 in cohort 3 (mean number of patients detected per month: 595).

**Figure 1:**
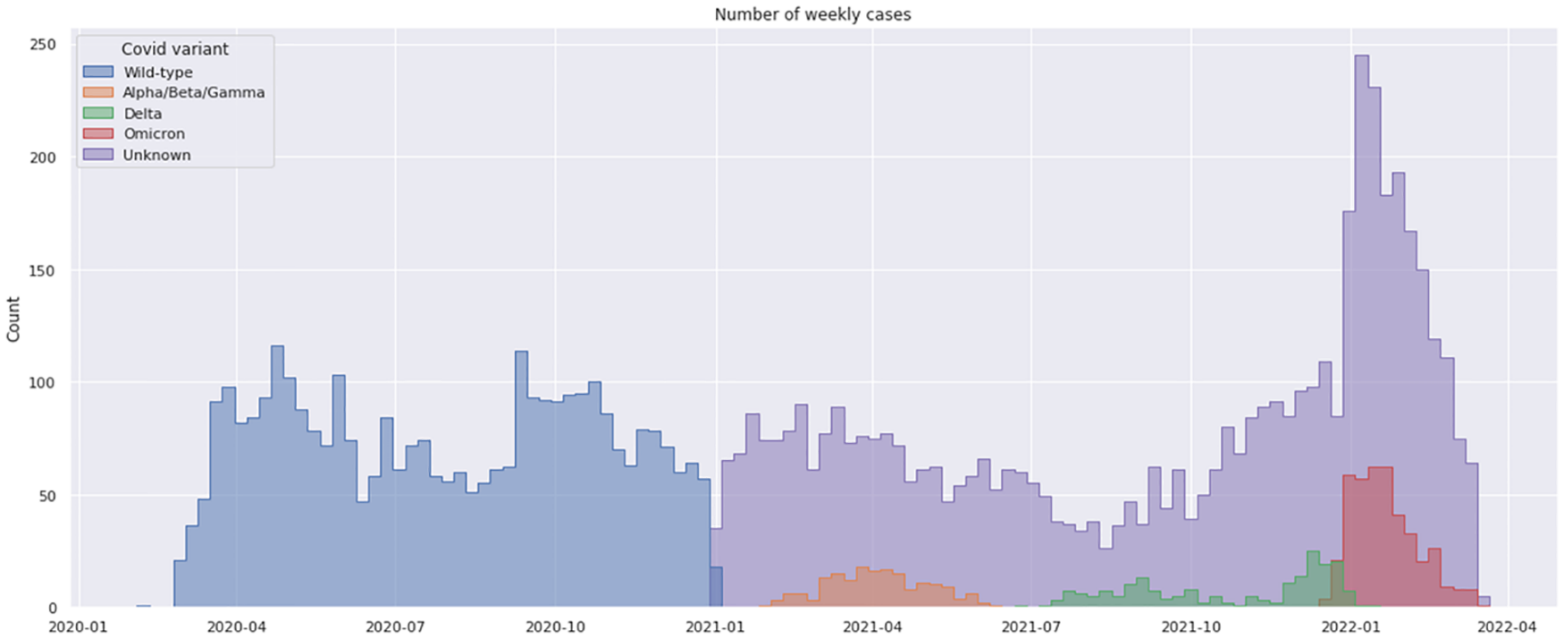
Weekly distribution of SARS-CoV2 cases variant genotyping in children hospitalized in the public hospital in the greater Paris area (AP-HP) from march 2020 to march 2022. The blue and violet bars represent the number of cases; the orange, green, and red bars represent the alpha-beta-gamma, delta, and omicron detected genotyping, respectively. For the first wave period (March 2020- Dec 2020) the genotyping was assigned to the historical variant (wild type).

Variant genotyping was automatically assigned to the historical variant for the forst wave period, while was available (the varian genotyping for 787 patients (9%) of the patients detected during the second and third waves (Figure 1). In more detail, in the second cohort, 92 patients presented with the emergent Delta variant cohort, 986 presented with the first historical variant cohort, and 176 patients presented with the Alpha/Beta/Gamma variant while the variant was not available for 2,607 patients. In the third cohort, 411 presented with the Omicron variant and 108 the Delta variant. The rest of the cohort was made up of patients with a non-determined variant.

Distribution according to age group is detailed in Table 1. Children between the age of 28 days and 5 years were the most represented in all periods. During the first wave, the least represented age group was patients younger than 28 days old (13.8%), whereas the proportion of this group increased during the other two surges (17.6% and 15.6%, respectively). Globally, the average patient's age decreased throughout the pandemic.

**Table 1:** Demography of the pediatric population analyzed.

		FIRST WAVE, MARCH 2020-SEPT 2020	SECOND WAVE, OCT 2020-OCT 2021	THIRD WAVE, NOV 2021-MARCH 2022	***P***-VALUE
**TOTAL**		2325	3861	2977	
**SEX (MALE)**		1 356 (58.3%)	2 131 (55.2%)	1 592 (53.5%)	2e–03
**AGE M ± SD**		6.1 ± 5.9	5.4 ± 6.0	4.4 ± 5.6	5e–24
**AGE**	**≤ 28D**	320 (13.8%)	681 (17.6%)	463 (15.6%)	3e–29
	**28D-5Y**	1 012 (43.5%)	1 798 (46.6%)	1 645 (55.3%)	
	**6-12Y**	542 (23.3%)	637 (16.5%)	472 (15.9%)	
	**13-18Y**	451 (19.4%)	745 (19.3%)	397 (13.3%)	

Sept: September; Oct: October; Nov: November. Age is expressed by mean (M) with standard deviation (SD). D: days; Y: years. Independence is tested with Chi-squared (*χ*^2^) analysis for categorical data, and one-way ANOVA for quantitative data.

### Study group characteristics

Characteristics of the three study groups are described in tables 2 to 4.

For study group 1, we collected data for PIMS and isolated myocarditis (PIMS/M) occurring after SARS-Cov2 infection (PIMS/M group) (Table 2). Focusing on this group, we observed that the highest number of these complications were registered during the first and second waves (incidence of 4.2%, 4.2%, and 2.4%, respectively, *p* = 3e-4). The PIMS/M patients were mostly within the age group of 5 to 12 years in all the three periods.

**Table 2:** Characteristics of pediatric patients hospitalized with pediatric inflammatory multisystem syndrome (PIMS) or acute myocarditis after SARS-Cov2 infection.

			First wave, March 2020-sept 2020	Second wave, Oct 2020-Oct 2021	Third wave, Nov 2021-March 2022	*p*-value
	TOTAL (*N*,%)		98, 4.2%	163, 4.2%	70, 2.3%	3e–04
**Demography**	Sex (male)		41 (41.8%)	104 (63.8%)	39 (55.7%)	3e–03
	Age m ± sd		8.4 ± 5.1	8.2 ± 4.6	8.3 ± 4.5	0.91
	Age	≤ 28d	0 (0.0%)	1 (0.6%)	0 (0.0%)	0.77
		28d–5y	33 (33.7%)	54 (33.1%)	23 (32.9%)	
		6–12y	42 (42.9%)	79 (48.5%)	36 (51.4%)	
		13–18y	23 (23.5%)	29 (17.8%)	11 (15.7%)	
**Medical history**	Heart disease		4 (4.1%)	10 (6.1%)	9 (12.9%)	0.07
	Chronic respiratory diseases		1 (1.0%)	4 (2.5%)	3 (4.3%)	0.40
	Immunological diseases		11 (11.2%)	8 (4.9%)	3 (4.3%)	0.09
	Cancer		0 (0.0%)	0 (0.0%)	0 (0.0%)	1.00
	Chronic kidney diseases		1 (1.0%)	6 (3.7%)	6 (8.6%)	0.04
	Obesity		2 (2.0%)	2 (1.2%)	2 (2.9%)	0.68
	Neurological and musculoskeletal disorders		0 (0.0%)	0 (0.0%)	1 (1.4%)	0.15
**Complications during the hospitalization**	Acute respiratory failure hypoxia		14 (14.3%)	4 (2.5%)	2 (2.9%)	2e–04
	Pneumonia		10 (10.2%)	4 (2.5%)	3 (4.3%)	0.02
	Length of stay m ± sd		8.6 ± 6.9	6.2 ± 5.0	6.1 ± 4.5	2e-03
	Admission to intensive care		79 (80.6%)	106 (65.0%)	40 (57.1%)	3e–03
	Death close to SARS-CoV-2 diagnosis		1 (1.0%)	1 (0.6%)	0 (0.0%)	0.70
**Treatment**	Non-invasive ventilation		27 (27.6%)	14 (8.6%)	1 (1.4%)	3e–07
	ECMO		1 (1.0%)	2 (1.2%)	0 (0.0%)	0.66
	Oxygen supply		22 (22.4%)	19 (11.7%)	4 (5.7%)	5e–03
	Inotropes/vasopressors		34 (34.7%)	34 (20.9%)	10 (14.3%)	5e–03

ECMO, extracorporeal membrane oxygenation.

The analysis of the clinical characteristics of this group of patients showed that respiratory distress occurred mostly during the first wave (14.3%) compared to the 2 other periods (2.5 and 2.9%, respectively). The third wave was characterized by a lower resort to active respiratory treatments (ventilator support, oxygen support, etc.) compared to the previous surges (i.e., non-invasive ventilation utilized for 27.6% of the patients during the first wave compared to 1.4% during the third wave). Only 57.1% of these patients were admitted to the intensive care (IC) department during the third wave while 80.6% and 65% were hospitalized in the IC unit during the first and second waves, respectively. The percentage of patients who needed inotrope support during the last SARS-Cov2 wave amounted to 14.3% compared to 34.7% of patients during the first wave.

The most represented underlying comorbidities in the personal medical history of pediatric patients presenting PIMS/M were immunologic diseases and cardiac diseases during the first wave (11.2% and 4.1%, respectively), while during the second and third waves, underlying cardiac diseases were the most represented comorbidities (6.1% and 12.9%, respectively), followed by immunologic diseases during the second wave (4.9%) and chronic kidney disease during the third wave (8.6%). Interestingly, during the third wave, the incidence of PIMS/M patients with a history of respiratory diseases increased compared to the other periods (4.3% vs. 1% and 2.5%).

Only two patients hospitalized in one of the AP-HP hospitals presenting PIMS/M died during the pandemic (during the first and second waves).

The number of patients hospitalized with a primary diagnosis of SARS-CoV2 infection (group 2) increased over time as 132 (5.6%), 308 (7.9%), and 442 (14.8%) patients were detected in the three periods of the pandemic (*p* = 1e-25), however, the specific respiratory symptoms decreased as well as admission to the intensive care department (Table 3).

**Table 3:** Characteristics of pediatric patients hospitalized with a principal diagnosis of SARS-CoV-2 infection.

			First wave, March 2020-sept 2020	Second wave, Oct 2020-Oct 2021	Third wave, Nov 2021-March 2022	*p*-value
	TOTAL (*N*,%)		132, 5.6%	308, 7.9%	442, 14.8%	1e–25
**Demography**	Sex (male)		65 (49.2%)	153 (49.7%)	248 (56.1%)	0.15
	Age m ± sd		7.2 ± 7.0	4.6 ± 6.2	3.3 ± 5.1	8e–11
	Age	≤28d	16 (12.1%)	59 (19.2%)	64 (14.5%)	3e–10
		28d—5y	51 (38.6%)	154 (50.0%)	285 (64.5%)	
		6–12y	23 (17.4%)	39 (12.7%)	52 (11.8%)	
		13–18y	42 (31.8%)	56 (18.2%)	41 (9.3%)	
**Medical history**	Heart disease		4 (3.0%)	8 (2.6%)	11 (2.5%)	0.94
	Chronic respiratory diseases		22 (16.7%)	45 (14.6%)	52 (11.8%)	0.27
	Immunological diseases		3 (2.3%)	8 (2.6%)	16 (3.6%)	0.62
	Cancer		5 (3.8%)	8 (2.6%)	14 (3.2%)	0.79
	Chronic kidney diseases		18 (13.6%)	18 (5.8%)	39 (8.8%)	0.03
	Obesity		2 (1.5%)	7 (2.3%)	4 (0.9%)	0.31
	Neurological and musculoskeletal disorders		2 (1.5%)	9 (2.9%)	13 (2.9%)	0.65
**Complications during the hospitalization**	Acute respiratory failure hypoxia		20 (15.2%)	16 (5.2%)	19 (4.3%)	2e–05
	Pneumonia		28 (21.2%)	37 (12.0%)	57 (12.9%)	0.03
	Length of stay m ± sd		8.6 ± 6.9	6.2 ± 5.0	6.1 ± 4.5	0.03
	Admission to intensive care		45 (34.1%)	63 (20.5%)	112 (25.3%)	1e–02
	Death close to SARS-CoV-2 diagnosis		5 (3.8%)	0 (0.0%)	1 (0.2%)	1e–05

The most represented cohort was pediatric patients with mild or asymptomatic SARS-CoV-2 infection. This third group was homogenously well represented in all the periods (Table 4). As in symptomatic SARS-Cov2 patients, most of the patients detected in this group were between the age of 28 days to 5 years old in the three periods. The proportional number of patients in this age group increased over time.

**Table 4:** Characteristics of pediatric patients hospitalized with mild or asymptomatic SARS-CoV-2 infection..

			First wave, March 2020-Sept 2020	Second wave, Oct 2020-Oct 2021	Third wave, Nov 2021-March 2022	*p*-value
	TOTAL (*N*,%)		2,095 (90.1%)	3,390 (87.8%)	2,465 (82.8%)	0.19
**Demography**	Sex (male)		1 250 (59.7%)	1 874 (55.3%)	1 305 (52.9%)	2e–05
	Age m ± sd		5.9 ± 5.8	5.3 ± 6.0	4.5 ± 5.6	1e–14
	Age	≤28d	304 (14.5%)	621 (18.3%)	399 (16.2%)	9e–22
		28d–5y	928 (44.3%)	1 590 (46.9%)	1 337 (54.2%)	
		6–12y	477 (22.8%)	519 (15.3%)	384 (15.6%)	
		13–18y	386 (18.4%)	660 (19.5%)	345 (14.0%)	
**Medical history**	Heart disease		41 (2.0%)	90 (2.7%)	68 (2.8%)	0.17
	Chronic respiratory diseases		371 (17.7%)	756 (22.3%)	434 (17.6%)	2e–06
	Immunological diseases		82 (3.9%)	142 (4.2%)	99 (4.0%)	0.87
	Cancer		104 (5.0%)	117 (3.5%)	114 (4.6%)	0.01
	Chronic kidney diseases		280 (13.4%)	366 (10.8%)	214 (8.7%)	3e–06
	Obesity		49 (2.3%)	75 (2.2%)	36 (1.5%)	0.06
	Neurological and musculoskeletal disorders		26 (1.2%)	73 (2.2%)	45 (1.8%)	0.05
**Complications during the hospitalization**	Acute respiratory failure hypoxia		30 (1.4%)	44 (1.3%)	43 (1.7%)	0.37
	Pneumonia		136 (6.5%)	211 (6.2%)	249 (10.1%)	2e–08
	Length of stay m ± sd		4.5 ± 7.2	4.5 ± 6.6	4.4 ± 5.7	0.75
	Death close to SARS-CoV-2 diagnosis		14 (0.7%)	16 (0.5%)	16 (0.6%)	0.56

The analysis of the patients with symptomatic or mildly symptomatic or asymptomatic SARS-Cov2 infection (groups 2 and 3) showed that 51 patients died within 5 days after the dismission. These deaths are likely due to other concurrent illnesses not related to the SARS-COv2 infection..

Despite the different clinical presentations of the SARS-CoV-2 infection, the incidence of concomitant diseases was similar in the symptomatic and mild or asymptomatic infected patients with higher involvement in patients with previous respiratory or chronic kidney diseases (Tables 3, 4).

Considering the whole population of SARS-Cov2 hospitalized pediatric patients (groups 2 and 3), the concomitant occurrence of pneumonia was higher during the third wave (10.2% during the third wave compared to 7% and 6.5% in the first and second waves).

## Discussion

To our knowledge, this study is one of the largest multicenter retrospective COVID-19 pediatric studies to date on more than 9,100 hospitalized pediatric patients presenting SARS-Cov2 infection in France. Using the AP-HP Covid Clinical Data Warehouse allowed an in-depth analysis of the clinical characteristics of these patients according to the period of hospitalization and the SARS-Cov2 variant identification, if available. The aim of this study was to characterize the impact of the SARS-Cov2 infection on the hospitalized pediatric population in the Great Paris area among the three different waves and, consequently, if possible, to analyze the link with the different subsequent SARS-Cov2 variants.

The greater Paris area has experienced five SARS-Cov2 infection surges (11): from March to May 2020, from September to November 2020, from March to May 2021, from July to September 2021, and from December 2021 to January 2022. For our analysis, we preferred to merge the periods characterized by a predominant SARS-Cov2 variant in order to better compare the impact and clinical characteristics in the pediatric population.

The spread of SARS-CoV-2 infection in the pediatric population, and in more detail, the incidence of the Omicron variant has been previously reported in the literature. Data from a pediatrics cohort in the United States suggested that hospitalization for symptomatic SARS-CoV-2 infection increased with the emergence of the Omicron variant, particularly in younger children (12). In another large cohort of pediatric patients in the United States (7,201 infected children), the incidence of SARS-COV2 with the Omicron variant predominated in children under the age of 5 years old (13). The REACT-1 cohort in England found a significantly higher incidence of the Sars-Cov2 Omicron infection in children (5 to 17 years old) compared to the previous variant infections (14).

Parts of these results are similar to our analysis where pediatric patients, less than 5 years old, are highly represented in the last period of the pandemic (71% of the 2,977 patients hospitalized during the third wave). The role of the incoming SARS-Cov2 vaccination for older children during the third wave is to be taken into account considering the higher incidence in very young patients (15).

In our study, the total number of pediatric patients hospitalized for symptomatic SARS-Cov2 infection increased over time (442 in the third wave vs. 132 and 308 in the first and second wave) with lung involvement, especially during the first period (21.2%). Nevertheless, considering the entire pediatric cohort (groups 2 and 3), a higher incidence of pulmonary involvement was observed during the third wave. Concomitant seasonal virus infections have to be considered in the analysis of the Omicron SARS-Cov2 infected pediatric patients (16) since SARS-CoV2 infection has similar symptoms to winter respiratory viruses. However, in our cohort, the symptoms could originate from several viruses without the possibility of attribution to SARS-Cov2. On the other hand, a recent study underlined that children without previous SARS-Cov2 immunization could present a more severe Omicron variant Covid-19 disease than children with previous immunization (17).

Interestingly, the analysis of the medical records showed that chronic respiratory diseases (i.e., asthma) were associated with a higher incidence of SARS-CoV2 infection. The association of chronic kidney diseases with SARS-Cov2 infection is probably due to a selection bias in patients in an exclusive hospital setting. Further studies need to focus on this population in order to state if chronic kidney disease patients are exposed to a higher risk of infection.

Pediatric Inflammatory Multisystem Syndrome correlated to SARS-Cov2 infection is an exclusive complication of COVID-19 infection in the pediatric population (18). These patients have been already well-characterized in the literature (19, 20). In our analysis, we showed that pediatric patients presenting PIMS or acute myocarditis following SARS-Cov2 infection were well represented in the three periods with higher incidence during the first and the second wave. Interestingly, in accordance with our results, a German study comparing the incidence of PIMS over the pandemic and the incidence of the different variants revealed a lower occurrence of PIMS correlating with the Omicron variant of SARS-COV2 infection (21). This is also concordant with other results from studies performed in the United Kingdom (22), Denmark (23), and Australia (24) which also found a lower incidence of PIMS during the dominant Omicron variant of SARS-CoV-2. This is probably due to the viral antigen changes and the subsequent host immune-inflammatory pathway activations. Further investigations is needed to complete these analyses since this complication could occur with a variable delay from the time of infection. Nevertheless, the diagnosis and management of PIMS patients improved over time and this is probably a significant reason why the severity of the disease seems to be less important during the last wave.

Several limitations of this study should be noted since some variables were not available in the database for the analysis, and potential inaccuracies can be present in the electronic health records. Although these findings should be interpreted with caution, this study illustrated the impact of the SARS-Cov2 infection on the hospitalized pediatric population providing valuable information on the characteristics and frequency over time. The robustness of the data analyzed rests on one of the largest multicenter retrospective SARS-Cov2 pediatric studies. Further analysis is needed to improve our knowledge of the new emerging variants and to monitor the frequency of reinfection.

## Data Availability

The raw data supporting the conclusions of this article will be made available by the authors, without undue reservation.
