# Supplementary material for: Analysis of the impact of the SARS-CoV-2 infection on the pediatric population hospitalized during the pandemic in the Greater Paris University Hospitals
Source: Front Pediatr. 2023 Feb 27;11:1044352. doi: 10.3389/fped.2023.1044352 (PMC10009109; doi:10.3389/fped.2023.1044352)
Supplement: Supplementary file 1 [file Table1.docx]

Supplementary Material

| **Supplemental Table 1. List of collaborators** | | |
| --- | --- | --- |
| **NOM Prénom** | **Affiliation** | **Contribution** |
| ANCEL Pierre-Yves | APHP Paris University Center | Local CDW coordinator |
| BAUCHET Alain | APHP Saclay University | Local CDW coordinator |
| BEEKER Nathanael | APHP Paris University Center | Data scientist |
| BENOIT Vincent | WIND Department APHP Greater Paris University Hospital | Data engineer |
| BERNAUX Mélodie | Strategy and transformation department, APHP Greater Paris University Hospital | Medical coordination of data analysis |
| BELLAMINE Ali | WIND Department APHP Greater Paris University Hospital | Data engineer, data scientist |
| BEY Romain | WIND Department APHP Greater Paris University Hospital | Data engineer, data scientist, regulatory assessment |
| BOURMAUD Aurélie | APHP Paris University North | Local CDW coordinator |
| BRÉANT Stéphane | WIND Department APHP Greater Paris University Hospital | Coordination of clinical research informatics |
| BURGUN Anita | Department of Biomedical Informatics, HEGP, APHP Greater Paris University Hospital | Medical & scientific coordination |
| CARRAT Fabrice | APHP Sorbonne University | Local CDW coordinator |
| CAUCHETEUX Charlotte | Université Paris-Saclay, Inria, CEA | Data integration and analysis |
| CHAMP Julien | INRIA Sophia-Antipolis – ZENITH team, LIRMM, Montpellier, France | Data integration and analysis |
| CORMONT Sylvie | WIND Department APHP Greater Paris University Hospital | Data standardisation |
| DANIEL Christel | "WIND Department APHP Greater Paris University Hospital; UMRS1142 INSERM" | Medical director of data standardisation and clinical research informatics |
| DUBIEL Julien | WIND Department APHP Greater Paris University Hospital | Data engineer |
| DUCLOS Catherine | APHP Paris Seine Saint Denis Universitary Hospital | Local CDW coordinator |
| ESTEVE Loic | SED/SIERRA, Inria Centre de Paris | Data engineer, data scientist |
| FRANK Marie | APHP Saclay University | Local CDW coordinator |
| GARCELON Nicolas | Imagine Institute | Data engineer, data scientist |
| GRAMFORT Alexandre | Université Paris-Saclay, Inria, CEA | Data engineer, data scientist |
| GRIFFON Nicolas | "WIND Department APHP Greater Paris University Hospital UMRS1142 INSERM" | Data standardisation |
| GRISEL Olivier | Université Paris-Saclay, Inria, CEA | Data engineer, data scientist |
| GUILBAUD Martin | WIND Department APHP Greater Paris University Hospital | Data engineer |
| HASSEN-KHODJA Claire | Direction of the Clinical Research and Innovation, AP-HP | Medical coordination of data-driven research |
| HEMERY François | APHP Henri Mondor University Hospital | Local CDW coordinator |
| HILKA Martin | WIND Department APHP Greater Paris University Hospital | Director of Big data platform |
| JANNOT Anne Sophie | Department of Biomedical Informatics, HEGP, APHP Greater Paris University Hospital | Biostatistician, local CDW coordonator |
| LAMBERT Jerome | APHP Paris University North | Local CDW coordinator |
| LAYESE Richard | APHP Henri Mondor University Hospital | Data scientist |
| LEBLANC Judith | Clincial Research Unit, Saint Antoine Hospital, APHP Greater Paris University Hospital | Data scientist |
| LEBOUTER Léo | WIND Department APHP Greater Paris University Hospital | Data engineer |
| LEMAITRE Guillaume | Université Paris-Saclay, Inria, CEA | Data engineer, data scientist |
| LEPROVOST Damien | Clevy.io | Data engineer, data scientist |
| LERNER Ivan | Department of Biomedical Informatics, HEGP, APHP Greater Paris University Hospital | Data engineer, data scientist |
| LEVI SALLAH Kankoe | APHP Paris University North | Data scientist |
| MAIRE Aurélien | WIND Department APHP Greater Paris University Hospital | Data engineer |
| MAMZER Marie-France | President of the AP-HP IRB | President of the AP-HP IRB |
| MARTEL Patricia | APHP Saclay University | Data scientist |
| MENSCH Arthur | ENS, PSL University | Data engineer, data scientist |
| MOREAU Thomas | Université Paris-Saclay, Inria, CEA | Data engineer, data scientist |
| NEURAZ Antoine | Department of Biomedical Informatics, HEGP, APHP Greater Paris University Hospital | Data engineer, data scientist |
| ORLOVA Nina | WIND Department APHP Greater Paris University Hospital | Data engineer |
| PARIS Nicolas | WIND Department APHP Greater Paris University Hospital | Data engineer, data scientist |
| RANCE Bastien | Department of Biomedical Informatics, HEGP, APHP Greater Paris University Hospital | Data engineer, data scientist |
| RAVERA Hélène | WIND Department APHP Greater Paris University Hospital | Data engineer |
| ROZES Antoine | APHP Sorbonne University | Data scientist |
| RUFAT Pierre | APHP Sorbonne University | Local CDW coordinator |
| SALAMANCA Elisa | WIND Department APHP Greater Paris University Hospital | Director of the Data & Innovation department |
| SANDRIN Arnaud | WIND Department APHP Greater Paris University Hospital | Director of the National Rare Diseases Database |
| SERRE Patricia | WIND Department APHP Greater Paris University Hospital | Data engineer, data standardisation |
| TANNIER Xavier | Sorbonne University | Data engineer, data scientist |
| TRELUYER Jean-Marc | APHP Paris University Center | Local CDW coordinator |
| VAN GYSEL Damien | APHP Paris University North | Local CDW coordinator |
| VAROQUAUX Gael | Université Paris-Saclay, Inria, CEA, Montréal Neurological Institute, McGill University | Data engineer, data scientist |
| VIE Jill-Jênn | SequeL, Inria Lille | Data engineer, data scientist |
| WACK Maxime | Department of Biomedical Informatics, HEGP, APHP Greater Paris University Hospital | Data engineer, data scientist |
| WAJSBURT Perceval | Sorbonne University | Data engineer, data scientist |
| WASSERMANN Demian | Université Paris-Saclay, Inria, CEA | Data engineer, data scientist |
| ZAPLETAL Eric | Department of Biomedical Informatics, HEGP, APHP Greater Paris University Hospital | Data engineer |

**Supplemental Table 2**

| **Medical history** | Heart disease | I30-I52, Q20-Q21-Q22-Q23-Q24-Q25-Q26 |
| --- | --- | --- |
|  | Chronic respiratory diseases | J30-J47 |
|  | Immunological diseases | R76, R834, R894, R874, R864, R844, R854, D80-D89; M30-M36; D59; K50; K51; M08-M09; L40 |
|  | Oncology | C, D0-D48 |
|  | Chronic kidney diseases | Q61-Q63, N |
|  | Obesity | E66 |
|  | Neurological and musculoskeletal disorders | G12-G13; Z74 |
|  | Invasive ventilation | GLLD015, GLLD008, GLLD004, GLLD009, GLLD005,ZZLB004 |
|  | Non-invasive ventilation | GLLD003, GLLD012, GLLD019 |
|  | ECMO | GLJF010,EQQP004 |
|  | Oxygen supply | GLLD017 |
|  | Inotropes/vasopressors | EQLF001, EQLF003 |
| **Complications during the hospitalization** | Acute respiratory failure hypoxia | J9600 |
|  | Pneumonia | J09-J18 |
| **Outcome** | PIMS or Myocarditis | U109; M303; I40; I41 |
|  | Myocarditis | I40; I41 |
|  | PIMS | U109; M303 |
|  | Diagosis Covid-19 | U071, B972, B342, U049 |
|  | Symptomatic Covid-19 | U0710, U0711, U0714, U0715 |
